# S-allylcysteine Improves Streptozotocin-Induced Alterations of Blood Glucose, Liver Cytochrome P450 2E1, Plasma Antioxidant System, and Adipocytes Hormones in Diabetic Rats

**DOI:** 10.5812/ijem.10927

**Published:** 2013-10-01

**Authors:** Ganapathy Saravanan, Ponnusamy Ponmurugan

**Affiliations:** 1 Department of Biochemistry, Centre for Biological science, K.S.Rangasamy College of Arts and Science, Thokkavadi, Tiruchengode,Tamil Nadu, India; 2 Department of Biotechnology, K.S.Rangasamy College of Technology, Thokkavadi, Tiruchengode, Tamil Nadu, India

**Keywords:** Antioxidants, Diabetes, Lipid Peroxidation, S- allylcysteine

## Abstract

**Background:**

S-allylcysteine, a garlic derivative, could have a protective effect against pathogenesis of diabetes mellitus.

**Objectives:**

Sustained free radical generation and oxidative damage to system leads to the final conclusion phase of diabetes and also it coexists with a constant diminution in the antioxidant status.The present study aims to evaluate the therapeutic effects of S-allylcysteine (SAC) against adipocytes hormones and antioxidant defense systems of plasma and erythrocytes of treptozotocin (STZ) induced diabetes in rats.

**Materials and Methods:**

Diabetic rats were administered SAC (150 mg/kg b.w) orally for 45 days. At 46^th^ day, the rats were anesthetized, and blood and liver sample were collected for analyzing glucose, plasma insulin, CYP2E1 activity, Thiobarbituric acid reactive substances (TBARS), hydroperoxide, enzymatic and nonenzymatic antioxidants, reduced glutathione (GSH), ceruloplasmin, plasma leptin, and adiponectin.

**Results;:**

The levels of glucose, CYP2E1 activity, Thiobarbituric acid reactive substances (TBARS), hydroperoxide, and ceruloplasmin were increased significantly; whereas, the levels of plasma insulin, reduced glutathione, enzymatic and nonenzymatic antioxidants, leptin and adiponectin were decreased in experimental diabetic rats. Administration of SAC to diabetic rats led to a decrease in the levels of glucose, CYP2E1 activity, TBARS, and ceruloplasmin. In addition, the levels of plasma insulin, enzymatic and nonenzymatic antioxidants leptin and adiponectin were increased in SAC treated diabetic rats. Gliclazide, a standard drug for diabetes, was used for the comparative purpose.

**Conclusions:**

The results of the present investigation suggest that SAC could be used as a food supplement in the treatment of diabetes characterized by provoked antioxidant status, altered blood glucose, and hormones level.

## 1. Background

Diabetic mellitus (DM) is a chronic metabolic disorder characterized by hyperglycemia which affects all the metabolic pathways (1). It is a systemic disease with relentless metabolic imbalances and pathological alterations in many tissues and premature mortality, costing for at least 10% of total healthcare expenses in many countries (2). The present number of diabetics across the world exceeds 150 million, which has been predicted to rise to >300 million by the year 2025 (2), and it might be due to an increase in sedentary lifestyle, consumption of energy rich diet, and obesity (3). The occurrence of diabetes in urbanized countries is rapidly increasing, and this pandemic disease is one of the most persistent causes of death in developed countries. According to recent studies in India, the number of diabetics wouldbe 84–224 million by the year 2025 and the most prevalence wouldbe among urban population (4).

Oxidative stress, the occurrence of oxidant factors plays a key role in the development of diabetes and its complications (5). Persistent hyperglycemia can enhance the oxidative stress by increasing glucose auto-oxidation, nonenzymatic protein glycation, and activation of polyol pathway (6). It is suggested that the rise in free radical activity may play an imperative role in lipid peroxidation and protein oxidation of cellular structures which are the main causes of morbidity and mortality in diabetes. In recent years, there has been increased interest in oxygen-reactive species generation and its role in the development of complications of diabetes. Recently, a growing concern has brought back to traditional and alternative medicines which replace the synthetic one. Results of the previous report had clearly stated that apart from traditional antidiabetic treatment, antioxidant therapy would be beneficial in diabetes (7).

Cytochrome P450 2E1 (CYP2E1), a source of reactive oxygen species (ROS), metabolizes different endogenous compounds, viz, fatty acids, lipid hydroperoxides, and ketone bodies (8). Ann et al. (9) reported that CYP2E1 is involved in the etiology and pathology of many diseases including diabetes (9). Previous report has shown that the expression of CYP2E1 messenger (m) RNA and protein is increased during diabetes (8). An increased CYP2E1 expression in various tissues of STZ-induced diabetic rats including the liver has been reported recently (10).During diabetes, the elevated CYP2E1 level may be an important risk factor for oxidative stress (9).

Persistent hyperglycemia is always associated with increased oxidative stress (7). During the course of diabetes, autoxidation of glucose and declined antioxidant enzymes system seem to be relevant to the elevated oxidative stress (11). Recent reports indicated that leptin and adiponectin were negatively modulated by oxidative stress (12, 13).

Recently, plant based treatment has been thought to be effective for the prevention and control of various diseases including diabetes (14). Garlic (Allium sativum, Liliaceae) is a rich source of bioactive compounds and is used in folk medicine for the treatment of various diseases. S-allylcysteine (SAC) a garlic derivative has been reported to have diverse potential including antioxidant activity (15), anticancer (16), antihepatotoxic (17), and neutrotrophic activity (18). Data from our recent study clearly reported the therapeutic potential of SAC against hyperglycemic status in diabetic rats (19). SAC also reverses the alterations in carbohydrate metabolizing enzymes (20), and glycoprotein metabolism (21).

## 2. Objectives

The present study was investigated to evaluate the therapeutic effect of SAC and gliclazide on oxidative stress induced by hyperglycemia, which enhances the modulations in erythrocytes and adipocytes hormones of STZ – induced diabetic rats. The effects produced by SAC were compared with gliclazide.

## 3. Materials and Methods

### 3.1. Animals

Adult male albino rats of Wistar strain weighing around 150 to 180g were used in this study. They were housed in polypropylene cages over husk bedding, and a 12 hour light and dark cycle was maintained throughout the experimental period. Rats were fed a commercial pellet diet and water ad libitum. The experiments were conducted according to the ethical norms approved by the Institutional Animal Ethics Committee guidelines.

### 3.2. Chemicals

SAC (99%) was purchased from LGC Prochem, Bangalore, India.Streptozotocin and all other chemicals (acids, bases, solvents and salts) used were of analytical grade obtained from Himedia, Bangalore, India. 

### 3.3. Animal Treatment and Induction of Diabetes

Type 1 diabetes was induced by the administration of STZ (55 mg/kg b.w, i.p). Blood glucose concentrations were measured at 72 h after injection of STZ. Rats with blood glucose level >250 mg/dl were used in the present study. Diabetic rats were divided into4 different groups, namely normal control rats, diabetic control rats, diabetic rats treated with SAC 150mg /kg b.w, p.o. daily[20], and gliclazide 5 mg/kg b.w p.o. daily(22) for 45 days.

Blood was collected at different time interval (10^th^, 20^th^, and 45^th^days) by nicking the tip of tail under light ether anesthesia in clean dry tubes. It was used for the estimation of glucose. At the end of 45 days treatment, animals were fasted for 10 hours and killed by cervical decapitation. Blood samples were collected for the estimation of antioxidant parameters.

### 3.4. Biochemical Assays

#### 3.4.1. Determination of Blood Glucose, Plasma Leptin and Adiponectin

Commercially available diagnostic kits (Sigma Diagnostics (I) Pvt. Ltd., Baroda, India) were used for the estimation of blood glucose, plasma leptin, and adiponectin. 

#### 3.4.2. Preparation of Haemolysate

For preparation of haemolysate, 2mL of blood was taken, and erythrocytes were separated by centrifugation at 1000×g for 10 min at 4^◦^C. The lysate was repeatedly washed with 10 mmol/L phosphate buffer saline (PBS), and adjusted to a hematocrit (HCT) of 5 or 10%. An aliquot of 0.5 ml washed red blood cells was lysed with 4.5 mL of ice cold distilled water to prepare haemolysate.

#### 3.4.3. Determination of Lipid Peroxidation

TBARS were estimated by the method of Fraga et al. (23). 0.5 mL of plasma was treated with 2 ml (1:1:1 ratio) TBA–TCA–HCl reagent (thiobarbituric acid, 0.37%, 0.25N HCl, 15% Trichloroacetic acid) and placed for 15 min in a boiling water bath and cooled. The clear supernatant was measured against reference blank at 535 nm, and the values were expressed as mM/dL. 

The level of hydroperoxide was determined by the method of Jiang et al. (24). 1.8 mL of Fox reagent was mixed with 0.2 mL of plasma, and then incubated for 30 min at room temperature, then read at 560 nm. The results were expressed as mM/dL.

#### 3.4.4. Determination of Vitamin C

Vitamin C was estimated by the method of Omaye et al. (25). Reduced dehydroascorbic acid was treated with 2, 4-dinitrophenylhydrazine (DNPH) to form the derivatives bis- 2,4-dinitrophenylhydrazone which undergoes rearrangement to form a product with an absorption maximum at 520nm.

#### 3.4.5. Determination of Vitamin E

Vitamin E was estimated by the method of Desai (26). The lipid residue was mixed with ferric chloride, orthophosphoric acid, and bathophenanthroline reagents. Vitamin E reduces ferric ion to ferrous ions and forms a pink colored complex with bathophenanthroline orthophosphoric acid. Absorption due to the pink complex was measured at 536nm.

#### 3.4.6. Determination of Reduced Glutathione

 Reduced glutathione was measured according to the method of Beutler and Kelley (27) (1963). The supernatant reacted with 5, 5’-dithio-bis-2-nitrobenzoic acid (DTNB), and the yellow derivativewas measured 412 nm. 

#### 3.4.7. Determination of Ceruloplasmin

 Ceruloplasmin was estimated by the method of Ravin (28). Ceruloplasmin oxidized p-phenylenediamine to form a purple colored compound. 1.0 mL of sodium azide solution was added to 0.05 mL of plasma. Then 1.0 mL p-phenylenediamine was added, mixed well and kept in a water bath at 37°C for one hour.All the tubes were kept at 4°C for 30 minutes.The color developed was read at 540 nm. 

#### 3.4.8. Determination of Superoxide Dismutase

Superoxide dismutase (SOD) activity was assayed by the method of Kakkar et al. (29). The method involves generation of superoxide radical and its detection by nitrite formation from hydroxylamine hydrochloride. The nitrite reacts with sulphanilic acid to produce a diazonium compound which subsequently reacts with naphthylamine to produce a red azo compound whose absorbance is measured at 543 nm.

#### 3.4.9. Determination of Catalase

Catalase levels were determined by the Aebi’s modified colorimetric method (30). 0.1 mL of tissue lysate and 0.4 mL of hydrogen peroxidewere added to 0.9 mL of phosphate buffer. After 60 seconds 2.0 mL of dichromate-acetic acid mixture was added. The tubes were kept in boiling water bath for 10 minutes, and the color developed was read at 620 nm.

#### 3.4.10. Determination of Glutathione Peroxidase

GPx activity was measured using the Paglia and Valentine’s method (31). 0.1 mL of sodium azide and 0.5 mL of sample were addedto 0.2 mL of tris buffer, 0.2 mL of EDTA. To this mixture, 0.2 mL glutathione followed by 0.1 mL hydrogen peroxide was added. The contents were mixed well and incubated at 37^o^C for 10 minutes along with a tube containing all the reagents except sample. After 10 minutes the reaction was arrested with the addition of 0.5 mL of 10 % TCA, centrifuged and the supernatant was assayed for glutathione.

#### 3.4.11. Determination of CYP2E1 Activity

Assay of CYP2E1 activity wasperformed based on Ahn et al. (9) with slight modification. Liver samples were homogenized (1:2, w/v) using 0.2 M phosphate buffer (pH 7.4) containing 2 mM EDTA. The pellets were then suspended in a 0.2M sucrose containing 1 mM EDTA and stored at -80˚C for further use. The enzyme activity was measured using the microsomal fractions, and the values were expressed as nmol hydroxylated nitrophenol/min/mg protein. The protein concentrations were estimated using folin phenol using bovine albumin solution as the standard.

### 3.5. Statistical Analysis

 All the results were expressed as Mean ± S. D. Data was statistically evaluated with SPSS\10.0 software. Hypothesis testing methods included one way analysis of variance (ANOVA) followed by least significant difference (LSD) test, P < 0.05 was considered to indicate statistical significance.

## 4. Results

**Figure 1.**
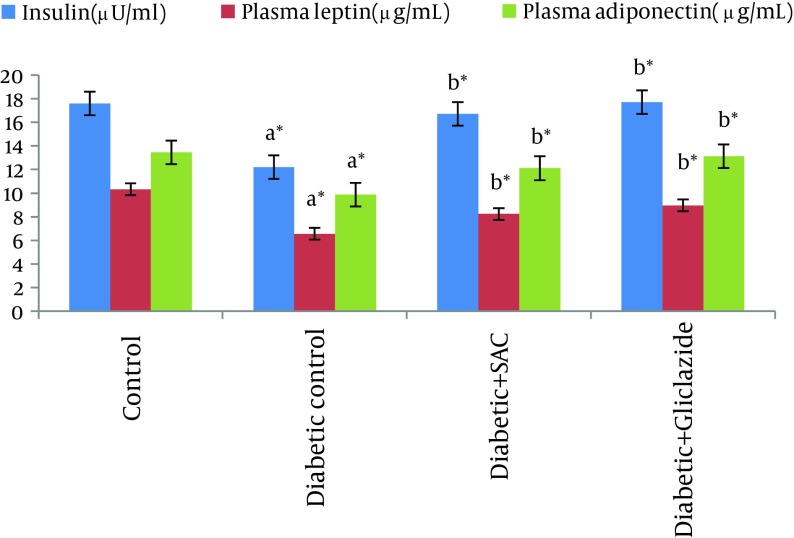
Effects of SAC and Gliclazide on Plasma Insulin, Leptin and Adiponectin(Values Are Mean ± SD, n = 6) ^a^ Group II compared with Group I ^b^ Group III and IV compared with Group II *P < 0.05

Table 1 and Figure 1 showed the level of blood glucose, plasma insulin, leptin and adiponectin in control and experimental animals.

The blood glucose was analyzed at different time intervals (10th, 20th and 45th day). The diabetic control rats showed a significant increase in the level of blood glucose with significant decrease in the level of plasma insulin, leptin and adiponectin. However, the level of blood glucose and plasma insulin, leptin and adiponectin werereturned to near normal concentrations in diabetic rats treated with SAC and gliclazide. 

**Table 1.** Effects of S-allylcysteine of Garlic on Blood Glucose Level at Different Intervals in Control and Experimental Diabetic Rats

Rat Groups	Initial, mg/dL	10^th^day, mg/dL	20^th^day, mg/dL	45^th^day, mg/dL
**Control**	95.6 ± 3.1	78.1 ± 3.6	105.6 ± 10.4	84.0 ± 16.1
**Diabetic control**	275.3 ± 10.9^a,b^	376.0 ± 31.7^a,b^	430.8 ± 84.0^a,b^	461.1 ± 28.0^a,b^
**Diabetic + S-allylcysteine (150mg/ kg body weight) **	268.3 ± 9.5^a,c^	320.3 ± 19.7^a,c^	205.5 ± 9.6^a,c^	105.0 ± 16.5^a,c^
**Diabetic +gliclazide (5 mg/ kg bodyweight)**	252.8 ± 74.0^a,c^	296.2 ± 13.7^a,c^	165.2 ± 14.8^a,c^	103.7 ± 10.8^a,c^

^a^Values are mean ± SD, n = 6, *P < 0.05

^b^Significantly different from untreated control

^c^Significantly different from diabetic control

Table 2 summarized the concentration of TBARS and hydroperoxides in plasma and erythrocytes of control and experimental rats. The levels of TBARS and Hydroperoxides were significantly increased in diabetic animals compared to control animals. Oral treatment of SAC and gliclazide tended to bring plasma TBARS and hydroperoxides towards near normal levels. 

**Table 2.** Effects of S-allylcysteine of Garlic on Thiobarbituric Acid Reactive Substances (TBARS) and Hydroperoxides in Plasma and Erythrocytes of Control and Experimental Diabetic Rats

Rat Groups	TBARS, Mean ± SD (n = 6)		Plasma Hydroperoxides, mM/dL, Mean ± SD (n = 6)
	Plasma, nmol/mL	Erythrocytes, pM/mg Hb	
**Control**	3.42 ± 0.54	1.71 ± 0.30	1.98 ± 0.92
**Diabetic control**	4.91± 0.84^a^	2.15 ± 0.10^a^	4.41 ± 0.97^a^
**Diabetic+ S-allylcysteine (150 mg/kg body weight)**	3.06 ± 0.79^c^	1.62 ± 0.20^c^	2.87 ± 0.21^b^
**Diabetic+gliclazide (5mg/kg body weight)**	2.59 ± 0.54^d^	1.69 ± 0.10^d^	2.59 ± 0.54^d^

^a^Significantly different from control P < 0.001

^b^Significantly different from diabetic control P < 0.05

^c^Significantly different from diabetic control P < 0.01

^d^Significantly different from diabetic control P < 0.001

Table 3 showed the plasma levels of vitamin C, vitamin E and also the GSH level in both plasma and erythrocyte of control and experimental rats. There was a significant decrease in the level of vitamin C, vitamin E and GSH in plasma of diabetic rats. Administration of SAC and gliclazide tended to bring the levels of nonenzymatic antioxidants towards near normal levels. 

**Table 3.** Effects of S-allylcysteine of Garlic on Nonenzymatic Antioxidants in the Plasma and Erythrocytes of Control and Experimental Diabetic Rats

Rat Groups	Plasma, mg/Dl, Mean ± S.D (n = 6)			Erythrocytes Reduced Glutathione, µmol/g Hb, Mean ± S.D (n = 6)
	Vitamin C	Vitamin E	Reduced Glutathione	
**Control**	5.53 ± 1.03	3.61 ± 0.51	29.33 ± 2.63	39.68±2.67
**Diabetic control**	3.07 ± 0.97^a,b^	1.91 ± 1.38^a^	20.14±1.11^a,b^	27.36±0.81^a,b^
**Diabetic + S-allylcysteine (150mg/ kg body weight)**	4.81 ± 0.60^c^	3.01 ± 0.59	25.95±3.07^c^	36.62±3.26^d^
**Diabetic +gliclazide(5 mg/ kg body weight)**	5.12 ± 0.32^c^	3.55 ± 0.96^e^	26.81±2.17^d^	38.7±2.11^d^

^a^Significantly different from control *P < 0.05

^b^Significantly different from control P < 0.001

^c^Significantly different from diabetic control P < 0.01

^d^Significantly different from diabetic control P < 0.001

^e^Significantly different from diabetic control P < 0.05

Table 4 exemplified the activities of SOD, CAT, GPx and ceruloplasmin in diabetic and control rats. There was a significant decrease in the activity of SOD, CAT and GPx and concomitant increasein the level of ceruloplasmin in diabetic rats when compared to control rats. Oral administration of SAC and gliclazide normalized the levels of enzymatic antioxidants towards normal. 

**Table 4.** Effects of S- Allylcysteine of Garlic on Superoxide Dismutase (SOD), Catalase (CAT), Glutathione Peroxidase (GPx) and Ceruloplasmin in Control and Experimental Diabetic Rats

Rat Groups ^a^	SOD, U^a^/mg Hb	CAT, U^b^/mg Hb	GPx, U^c^/mg Hb	Ceruloplasmin, mg/dL
**Control**	7.26 ± 0.58	133.4 ± 6.04	14.85 ± 2.22	23.2 ± 3.16
**Diabetic control**	3.7 ± 0.76^b^	90.68 ± 1.88^b^	9.68 ± 0.59^b^	38.79 ± 5.8^b^
**Diabetic + S-allylcysteine (150mg/ kg body weight)**	5.07 ± 0. 53^c^	122.4 ± 5.69^e^	13.97 ± 1.22^d^	27.1 ± 6.67^d^
**Diabetic + gliclazide** **(5 mg/ kg bodyweight)**	5.48 ± 0 .85^d^	127 ± 8.9^e^	14.31 ± 1.94^e^	26.65 ± 5.49^d^

U^a^The amount of enzyme required to inhibit 50% NBT reduction

U^b^Micromoles of H2O2 utilized/ min

U^c^µmole of glutathione oxidized per min per mg of protein

^a^Values are mean ± SD, n ± 6

^b^Significantly different from control P < 0.001

^c^Significantly different from diabetic control P < 0.05

^d^Significantly different from diabetic control P < 0.01

^e^Significantly different from diabetic control P < 0.001

**Figure 2.**
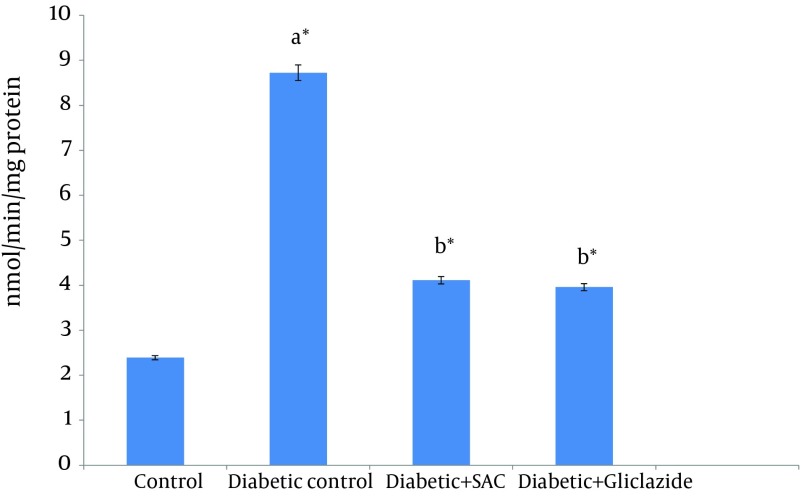
Effects of SAC and Gliclazide Supplementation on CYP2E1 Activity in the Liver of Control and Experimental Groupsof Rats (Values Are Mean ± SD, n = 6) a Group II compared with Group I b Group III and IV compared with Group II *P < 0.05

CYP2E1-catalyzed p-nitrophenol hydroxylation activity in the liver of experimental and control rats were showed in Figure 2. Due to STZ intoxication, CYP2E1 activities increased in the liver of control and experimental group of rats. SAC administration to diabetic rats decreased the CYP2E1 activity near to normal. 

## 5. Discussion

Hyperglycemia induced by streptozocin in animals is considered to be a good model for the preface screening of drugs active against diabetes (32). ß-cells of pancreas are particularly liable to be damaged by STZ which leads to hyperglycemic condition (33). In the present investigation, the diabetic rats were found to have higher glucose levels and lower level of insulin when compared to normal control rats. From the results of the present experiment, it was observed that the daily administration of SAC during 45 days decreased the blood sugar in STZ induced diabetic rats. It is perhaps due to stimulation of insulin secretion from remnant pancreatic β– cells, which in turn enhances glucose utilization by peripheral tissues of diabetic rats either by promoting glucose uptake and metabolism, or by inhibiting hepatic gluconeogenesis (19). 

Leptin and adiponectin, the chemical messengerssecreted by adipocytes, contribute to the regulation of lipid and glucose metabolism (34, 35). Leptin acts to reduce food intake and increase energy expenditure by activating specific hypothalamic receptors. Adiponectin improves insulin sensitivity by inspiring glucose uptake and fatty acid oxidation in skeletal muscle (36). In the present study, the plasma leptin and adiponectin levels were found to be lowered in the diabetic control rats compared tothe control rats. The results of our study are similar with the results of Thule et al. (37) and Kosova et al. (38) who found that plasma leptin and adiponectin were reduced in streptozocin-induced diabetic rats. In contrast to our results, some authors reported that plasma leptin and adiponectin were increased in patients with type 1 diabetes (39-41). The reasons for this discrepancy are not clear. From these results, the decreased level of plasma leptin and adiponectin levels in insulin-dependent diabetes mellitus may well be caused by an insulin deficiency and/or increased lipolysis. Oral administration of SAC to diabetic rats led to an increase in Leptin and adiponectin levels. These observations suggest that the increased plasma leptin and adiponectin levels after SAC supplementation might be attributable to increased insulin level and decreased lipolysis (42). 

TBARS assessment in plasma helps to assess the extent of tissue damage (43). In the present study, we found an increase in the levels of plasma hydroperoxide and TBARS which is a key factor of lipid peroxidation.The major pathological outcome of membrane lipid peroxidation by free radical induction includes increased membrane rigidity, decreased cellular deformability, reduced erythrocyte survival, and lipid fluidity (44, 45). Tremendous increase in lipid peroxidation observed in diabetic rats is attributed to constant hyperglycemia which causes increased production of reactive oxygen species (ROS). This may be due to the oxidation of monosaccharide (46) which causes tissue damage in membranes (47). Oral treatment with SAC prevented the increased level of lipid peroxidation markers, which could be as a result of improved glycemic control.

Vitamin C is a venerable water-soluble hydrophilic antioxidant which principally scavenges free radicals. It disappears faster than other antioxidants on exposure to reactive oxygen species (48). Vitamin C contributed up to 24% of the total peroxyl radical-trapping antioxidant activity (TRAP) (49). We have noted a decreased level of plasma vitamin C in the diabetic rats. This could be due to the increased consumption of vitamin C in the deactivation of the increased levels of reactive oxygen species or to the decreased GSH level, since the GSH is necessary for the recycling of vitamin C (50). Administration of SAC and gliclazide to diabetic rats tends to bring the Vitamin C to near normal levels, which could be as a result of increased GSH level.

Vitamin E, an imperative free radical scavenging chain-breaking antioxidant within biomembrane (51) interrupts the chain reactions of lipid peroxidation by reacting with lipid peroxyl radicals, thus defending the cell structures against damage (52). The amplified oxidative stress which accompanies a decrease in the level of antioxidants leads to decreased level of α-tocopherol in diabetics (53). Treatment with SAC and gliclazide brought Vitamin E to near normal levels which could be as a result of decreased membrane damage.

Glutathione (GSH) is a metabolic regulator and putative indicator of health. GSH also functions as a free radical scavenger and in the repair of free radical caused biological damage (54). A marked depletion in the GSH content in plasma was observed in diabetic control rats. Reduced level of GSH in the circulation during diabetes represents its increased utilization due to oxidative stress (55). Hence, drugs that prevent the generation of these oxygen free radicals or increase the free radical scavenging enzymes may be effective in STZ induced diabetes. Furthermore, SAC treatment showed a significant renovation in GSH content of diabetic rats which may be due to the antioxidant property of SAC. 

GPx plays a central role in the catabolism of H2O2 and the detoxification of endogenous metabolic peroxides and hydroperoxides which catalyzes GSH (56). The decreased activity of GPx in this study might be due to the lowered level of GSH (57) in diabetic state. A marked increase in GPx was observed in diabetic rats treated with the SAC. This might reflect the antioxidant potency of SAC which reduced glucose levels and prevented glycation and inactivation of GPx. Thus GPx activity was induced to scavenge free radicals in diabetic rats.

Ceruloplasmin is a copper containing oxidase which inhibits lipid peroxidation by binding with the copper (58). During diabetes, the ceruloplasmin level is increased due to the generation of superoxide radicals and hydrogen peroxide (59). The level of ceruloplasmin was significantly increased in diabetic rats when compared to control rats which may facilitate the scavenging action on peroxyl radicals (60). Treatment with SAC prevented the increased level of ceruloplasmin compared to diabetic control. In SAC treated diabetic rats, low plasma ceruloplasmin levels might be due to an increase in their utilization to neutralize free radicals.

SOD, an essential defense enzyme catalyzed the dismutation of superoxide radicals (61). Due to hyperglycemia, the autoxidation of glucose results in the formation of hydrogen peroxide which inactivates SOD (62). Therefore, the accumulation of H2O2 may be one of the explanations for decreased activity of SOD in diabetic condition. SOD is accountable for the detoxification of deleterious oxygen radicals (63). The observed decrease in SOD during diabetes could result from inactivation by H2O2 or by glycation of the enzyme, which have been reported to occur in diabetes (64). In this study, the oral supplementation of SAC and gliclazide maintained the SOD activities at near control level. Namely, our results indicated that the preventive effects of SAC may be due to inhibition of lipid peroxidation and scavenging of free radicals by its antioxidant nature.

Catalase is a haem containing ubiquitous enzyme which removes toxic free radicals in vivo. CAT reduces H2O2 produced by dismutation reaction and prevents generation of hydroxyl radicals (65). The present study revealed that CAT activities were significantly inhibited in erythrocytes of diabetic group which could be due to increased oxygen metabolites causing a decrease in the activity of the antioxidant defense system. Further, it was suggested that decreased antioxidant enzyme activity in DM is due to nonenzymatic glycosylation of the enzymes (66). Treatment with SAC and gliclazide has reversed the CAT activities, which could be as a result of decrease in the levels of lipid peroxidation and or decreasing utilization for scavenging free radicals.

STZ intoxication alkylates DNA and causes induction of CYP2E1 protein and mRNA (67). Incomplete reduction of oxygen due to leakage of electrons through the electron transport chain leads to the excessive production of ROS catalyzed by CYP2E1. The elevated ROS production, lipid peroxidation and the lowered antioxidant defense are common in diabetes (7). In the present study, increased liver CYP2E1 activity was observed in diabetic control rats. SAC supplementation leads to decreased blood glucose level and increased plasma insulin level in diabetic rats. Lower body glucose concentration ameliorated the induction of CYP2E1 and associated with liver injury (7).

In conclusion, the present study demonstrated that SAC administration significantly improved glycemic, antioxidant status and adipocytes hormones in diabetic rats by enhancing insulin. In conclusion, our result confirmed the antidiabetic action of SAC, and showed the favorable effect of SAC on erythrocyte antioxidants defense system and adipocytes hormones in addition to its antidiabetic effect. 
